# Fibrinogen-like protein 2 expression correlates with microthrombosis in rats with type 2 diabetic nephropathy☆

**DOI:** 10.1016/S1674-8301(11)60015-8

**Published:** 2011-03

**Authors:** Guanhua Su, Kun Liu, Yan Wang, Jue Wang, Xiaowei Li, Wenzhu Li, Yuhua Liao, Zhaohui Wang

**Affiliations:** a Department of Cardiology, Union Hospital, Tongji Medical College, Huazhong University of Science and Technology, Wuhan 430022, Hubei, China; b Division of Clinics, Tongji Medical College, Huazhong University of Science and Technology, Wuhan 430030, Hubei, China

**Keywords:** fibrinogen-like protein 2, microthrombosis, type 2 diabetes, diabetic nephropathy, tumor necrosis factor-α

## Abstract

Fibrinogen-like protein 2 (fgl2), a novel prothrombinase, is involved in microthrombosis. We examined fgl2 expression in the glomerular and tubulointerstitial capillaries and its correlation with microthromsis in rats with streptozocin-induced type 2 diabetic nephropathy. Our RT-PCR and immunoblotting analysis showed that fgl2 mRNA and protein levels were increased in microvascular endothelial cells of the glomeruli and renal interstitia at week 19 and became significantly elevated with the development of diabetic nephropathy (*P* < 0.01). Fgl2 was not or only weakly expressed in the renal tissues of normal rats. Furthermore, a direct significant correlation (*r* = 0.543, *P* < 0.01) was found between fgl2 expression and microthrombotic capillaries in the renal tissues. Enzyme linked immunosorbent assays (ELISA) additionally showed that circulating TNF-α levels in rats with type 2 diabetes were significantly elevated and closely correlated with fgl2 expression (*r* = 0.871, *P* < 0.01). Our results suggest that fgl2 may activate renal microthrombosis, thus contributing to glomerular hypertension and renal ischemia.

## INTRODUCTION

Diabetic nephropathy is the primary cause of chronic kidney disease (CKD) and end-stage renal disease (ESRD) in adults or the elderly and accounts for 30%-35% of patients on renal replacement therapy in North America[1]. Diabetic nephropathy complicates both type 1 and type 2 diabetes. However, most ESRD patients have type 2 diabetes because of the greater prevalence of type 2 diabetes worldwide (90% of all individuals with diabetes). Nevertheless, the pathophysiology, clinical features and morphology of diabetic nephropathy appear similar between type 1 and type 2 diabetes.

Diabetic nephropathy is characterized clinically by microalbuminuria and proteinuria and exhibits features of microangiopathy. Like other microvascular complications, diabetic nephropathy is chiefly attributed to chronic hyperglycemia[2],[3]. In addition, glomerular hypertension and hyperfiltration insulting glomerular filtration barrier are also involved in the pathogenesis[2],[4],[5]. As yet, the underlying pathophysiological mechanisms remain incompletely defined. In light of the evidence that microthrombosis contributes to capillary obliteration and retinal ischemia of diabetic individuals[6],[7], microthrombosis may play a role in diabetic microangiopathy.

Over the last decade, a novel prothrombinase fibrinogen-like protein 2 (also termed fgl2 or fibroleukin) has been demonstrated to act as a pivotal player in microthrombosis. Fgl2 is a direct prothrombinase that generates thrombin from prothrombin to initiate microthrombosis independent of the classic coagulation cascade[8],[9]. In viral hepatitis[10], acute vascular xenograft rejection[6],[11] and cytokine-induced fetal loss syndrome[12],[13], microthrombosis due to fgl2 is responsible for microvascular disturbance, resulting in ischemic injuries. However, little knowledge is available about fgl2 in diabetic nephropathy.

Diabetic nephropathy is usually diagnosed on clinical grounds without a renal biopsy. Thus, unlike primary renal diseases, it is not easy to obtain direct pathological data from diabetic individuals. In this study, we adopted an established rat model of type 2 diabetes[14] to examine the expression of fgl2 in endothelial cells of the glomerular and tubulointerstitial capillaries and investigated the possible significance of fgl2 in diabetic nephropathy.

## MATERIALS AND METHODS

### Type 2 diabetic nephropathy rat model

A total of 68 adult male SD rats weighing 150-200 g obtained from the Centre of Experimental Animals, Tongji Medical College, Huazhong University of Science and Technology. were randomly divided into the model group (*n* = 44) and the normal control group (NC group, *n* = 24). During the initial 10 w of the experiment, 44 rats in the model group were fed a high-calorie diet containing 18% fat, 20% sucrose, 2% cholesterol, 1% pig sodium cholate and 59% normal chow to induce insulin resistance. Following intraperitoneal injection of 30 mg/kg streptozocin, 24 of 44 rats in the model group developed type 2 diabetes as confirmed by plasma glucose levels. Twenty-four rats as controls were fed a normal chow diet and given the injection of the same volume of citrate buffer. Proteinuria was not stably present in diabetic rats until the 19^th^ week of the experiment. At the 19^th^, 23^rd^ and 28^th^ week of the experiment, 8 rats were sacrificed to observe microthrombosis and fgl2 expression, respectively. All procedures were carried out in accordance with the Guide for the Care and Use of Laboratory Animals (NIH, Pub.No.85-23, revised 1996).

### Sample collection and measurement of biochemical markers

Before rats were sacrificed at the 19^th^, 23^rd^ and 28^th^ week of the experiment, they were put into metabolic cage and fasted overnight. Urine was collected for 24 h, centrifuged to remove sediments, and then stored at -70°C for detection of urine protein. Blood was collected and measured for levels of serum creatinine and blood urea nitrogen (BUN). The right kidney was obtained and put into liquid nitrogen and stored for RT-PCR analysis. The left kidney was removed and fixed for subsequent immunohistochemistry analysis and microscopic observation.

### Histological analysis

The left renal tissue was fixed in 4% paraformaldehyde, embedded in paraffin, and then sectioned. Periodic acid-Schiff (PAS) and Masson staining of renal tissues were performed to observe renal histology and microthrombosis. Five random visual fields were selected from each section and analyzed by the HMIAS-2000 Imaging System (Wuhan Champion Image Technology Co., Ltd., China). Each field selected two glomeruli for observation. The proportion of renal tissue with microthrombosis per unit area in the glomeruli was calculated in each field at a magnification of 200× (*n* = 5).

### RT-PCR analysis

Total RNA was extracted from the kidney using TRIZOL reagent according to the protocol provided by the manufacturer (TIAGEN Biotechnology Co., Ltd., China). Oligonucleotide primers for fgl2 were synthesized as follows: (sense) 5′-GTCGCTCCAACTGGTAAAT-3′ and (anti-sense) 5′-AGGTCCCACTGCTTCTCTT-3′. As an internal control, the primers for GAPDH were as follows: (sense) 5′-CTATCGGCAATGAGCGGTTC-3′ and (anti-sense) 5′- CTTAGGAGTTGGGGGTGGCT-3′. The cDNA of fgl2 was obtained by reverse transcription from the total RNA in first strand synthesis system for RT-PCR kit (TaKaRa, Japan). The cDNA was amplified by polymerase chain reaction (PCR) with primers as described previously to detect the levels of fgl2 mRNA. After initial pre-denaturation at 94°C for 2 min, amplification was performed in a DNA thermocycler for 35 cycles (denaturation at 94°C for 30 s, annealing at 55°C for 30 s, and extension at 72°C for 60 s). A final extension of 5 min at 72°C completed the PCR. The PCR products were analyzed by electrophoresis on 2.0% agarose gels containing 0.5 mg/mL ethium bromide. Amplification of the fgl2 and GAPDH cDNA yielded products 204 and 706 bp in size, respectively. Levels of fgl2 mRNA were normalized against GAPDH by densitometric analysis.

### Immunohistochemistry analysis

The protein expression of fgl2 in rat kidney was determined by the immunohistochemical streptavidin-peroxidase (S-P) technique. Paraffin sections were routinely prepared. Briefly, the specimens were incubated with a 1:400 dilution of rabbit anti-rat fgl2 polyclonal antibodies (Beijing Biosynthesis Biotechnology, China) at 4°C overnight. Subsequently, sections were reincubated with biotinylated secondary antibodies of goat anti-rabbit IgG at 37°C for 20 min followed by 10 min incubation in an S-P complex solution. Finally, peroxidase activity was visualized with diaminobenzidine (DAB), counterstained with hematoxylin, dehydrated and mounted for microscopic examination. Negative control was set in the experiment. Five locations containing glomeruli and tubules were selected randomly from each section for analysis.

### Western blotting assays

Briefly, total extracts from renal tissues (20-50 µg protein per lane) were loaded onto 12% SDS-polyacrylamide gels. After the proteins were separated, they were transferred to nitrocellulose membrane. The membrane was blocked and probed with a polyclonal antibody against fgl2 (Beijing Biosynthesis Biotechnology, China) at a dilution of 1:200 in 5% nonfat milk in TBS. After washing with TBST and 0.5% Tween-20, the blot was incubated with secondary antibodies (Santa Cruz Biotechnology) conjugated to horseradish-peroxidase for 2 h at room temperature. Immunoreactive bands were detected with enhanced chemiluminescence reagent (Pierce Biotechnology). Protein expression levels were determined by densitometric analysis using NIH Image Version 1.61. Protein expression levels were normalized against β-actin.

### Circulating TNF-α assay

Circulating TNF-α concentration was assayed by enzyme-linked immunosorbent assay (ELISA) following the procedure recommended by the manufacturer (Wuhan USCN Sciences Corporation, China).

### Statistical analysis

Data were expressed as mean±SE. Statistical analysis was performed using SPSS 12.0 software. Differences between groups were analyzed by *t* test or ANOVA. All comparisons were specified in the figure legends and were performed between samples from the same experiment and time-point to check for statistical significance. A *P*-value less than 0.05 was considered statistically significant. Pearson's correlation coefficient (*r*) was used to quantify the strength of association between two continuous variables.

## RESULTS

### Levels of biochemical variables elevated in type 2 diabetic rats

**Fig. 1:**
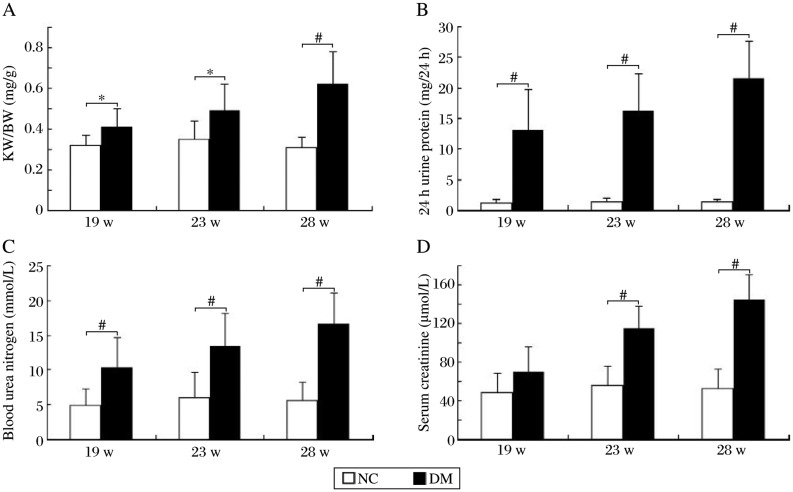
Renal hypertrophic index and biochemical variables of rats over time. A: Kidney weight to body weight ratios (KW/BW). B: levels of 24 h urine protein. C: levels of blood urea nitrogen (BUN). D: levels of serum creatine (SCr). *P < 0.05 and ^#^*P* < 0.01 compared to the normal control (NC) group.

On the 19^th^ week of the study, renal hypertrophic index calculated by kidney weight to body weight ratios (KW/BW), levels of 24 h urine protein and blood urea nitrogen (BUN) in type 2 diabetic rats were significantly higher than those of normal rats, whereas there was no significant difference in serum creatine content between the two groups. In the following weeks, KW/BW and levels of 24 h urine protein and BUN in type 2 diabetic rats were aggravated in line with serum creatine contents (***Fig. 1***).

### Histopathologic examinations of renal tissues

**Fig. 2:**
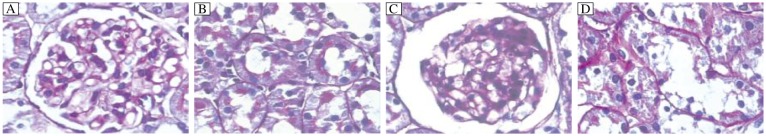
Histological analysis of renal tissues from rats (PAS,×400). A and B: Thickened glomerular basement membrane, mesangial matrix proliferation, glomerular capillary expansion, and vacuolar degeneration of renal tubule epithelial cells of diabetic rats were observed in diabetic rats. C and D: The changes described previously were not seen in normal rats.

**Fig. 3:**
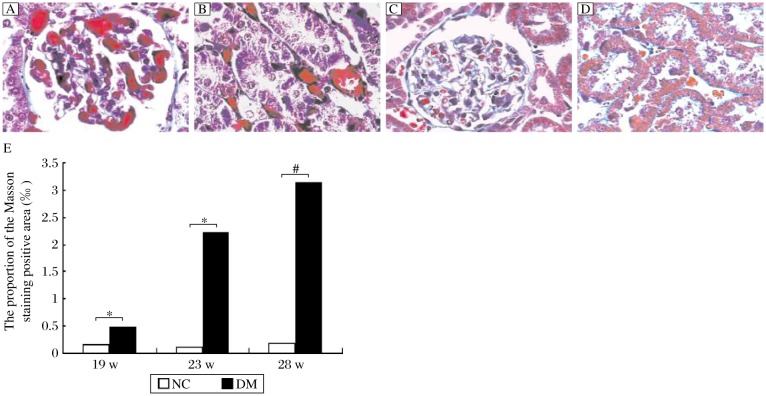
Histological analysis of renal tissues of rats (Masson staining, ×400). A. Proliferation of glomerular basement membrane, dissolution of the glomerular mesangium, and microthrombosis of glomerular capillary were observed in diabetic rats in the 28^th^ week of the experiment. B. Granular degeneration and vacuolar degeneration were seen in the renal tubule epithelial cells. C and D: Some red blood cells were seen in glomerular capillaries while no microthrombus was detected in controls. E: The proportion of the Masson staining positive area of the glomeruli per unit area (‰). *P < 0.05 and ^#^*P* < 0.01 compared to the normal control (NC) group.

Enlarged glomeruli, irregular thickened basement membrane (GBM) and obviously widened mesangial regions were observed in type 2 diabetic rats, accompanied by substantially increased amount of PAS positive materials in the mesangial regions. Meanwhile, mesangial cell growth and matrix accumulation were enhanced, which exhibited as moderate to severe proliferation as the course of the disease prolonged (***Fig. 2A*** and ***2B***). Microthrombosis was observed in parts of the glomerular capillaries. Protein casts appeared in some renal tubules, while vacuolar degeneration of the renal tubule epithelial cells, cellular infiltrates and thrombosis in some microvessels of the renal interstitium were observed (***Fig. 3A*** and ***3B***). In the normal rats, no obvious pathological changes were found in the glomeruli and renal tubules (***Fig. 2C*** and ***2D***). Meanwhile, no microthrombus was detected (***Fig. 3C*** and ***3D***).

MASSON staining of microthrombi in glomerular capillaries and renal intersitial micrangium of diabetic rats showed bright red regions. Those microthrombi connected tightly with the endothelium of capillaries or microvessels, which indicated that those were thrombi in situ in microvessels of diabetic rats and were composed of fibrins. The proportion of the MASSON staining positive area of glomeruli per unit area (‰) of the diabetic rats was significantly higher than that of the normal rats at all time points (***Fig. 3E***).

### Elevated expression of fgl2 protein and mRNA in diabetic rats

**Fig. 4:**
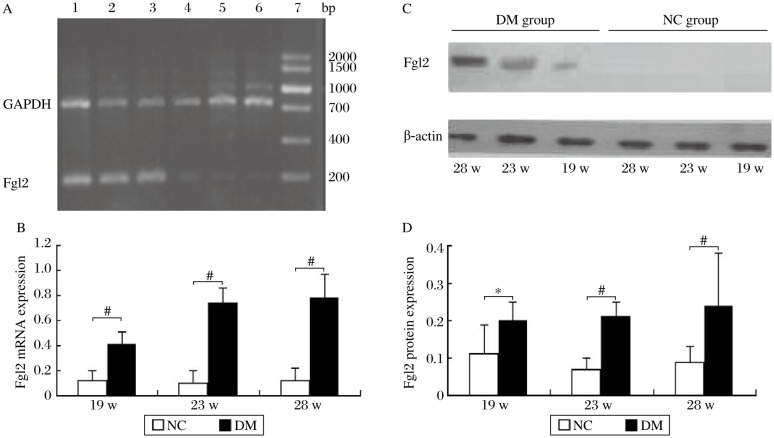
Quantification of fgl2 protein and mRNA expression in the diabetic and normal rats (*n* = 8). A: Agarose gel electrophoresis of *fgl2* and *GAPDH* mRNA amplification products. Lane 1-3 and Lane 4-6 represent the mRNA expression in the 19^th^, 23^rd^ and 28^th^ week of the diabetic (DM) group and the normal control (NC) group, respectively. Lane 7: DNA maker. B: *Fgl2* mRNA expression of renal tissue. C and D: Fgl2 protein expression in renal tissue by Western blotting analysis. *P < 0.05 and ^#^*P* < 0.01 compared to the NC group.

**Fig. 5:**
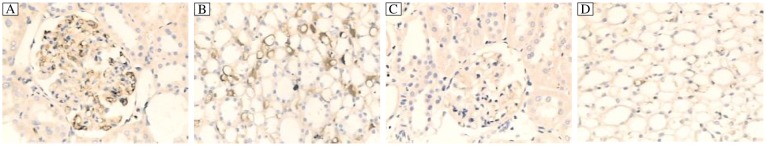
Typical expression of fgl2 in the renal tissue of rats (IHC,×200). Fgl2 was expressed in the microvascular endothelial cells of the glomerulus (A) and renal interstitium (B) in the diabetic rats at the 28^th^ week. No expression of fgl2 was observed in the glomerulus (C) and renal interstitium (D) of normal rats.

Fgl2 mRNA expression in the renal tissue of the diabetic rats was significantly higher than that of the even-aged rats in the NC group (***Fig. 4A, 4B***). Western blot analysis revealed that fgl2 protein expression in the diabetic rats began to increase from the 19th week during the development of diabetic nephropathy and was significantly elevated compared to the normal rats (***Fig. 4C, 4D***). Fgl2 was not or only weakly expressed in the renal tissue in normal rats, while it was markedly expressed on the microvascular endothelial cells of the glomeruli and renal interstitium in the diabetic rats (***Fig. 5***). Positive staining was also observed in the cytoplasm of some glomerular epithelial cells (***Fig. 5***).

### Up-regulation of fgl2 expression contributed to renal microthrombosis

A univariate correlation analysis was performed to quantify the strength of association between levels of fgl2 expression and the proportion of positive tissue with microthrombosis of the glomerulus per unit area. A significant direct correlation (*r* = 0.543, *P* < 0.01 by Pearson correlation analysis) was found between the average optical density value of fgl2 expression in the diabetic rats and the vessels with microthrombosis.

### Correlation between circulating TNF-α levels and fgl2 expression in the kidneys of diabetic rats

**Fig. 6:**
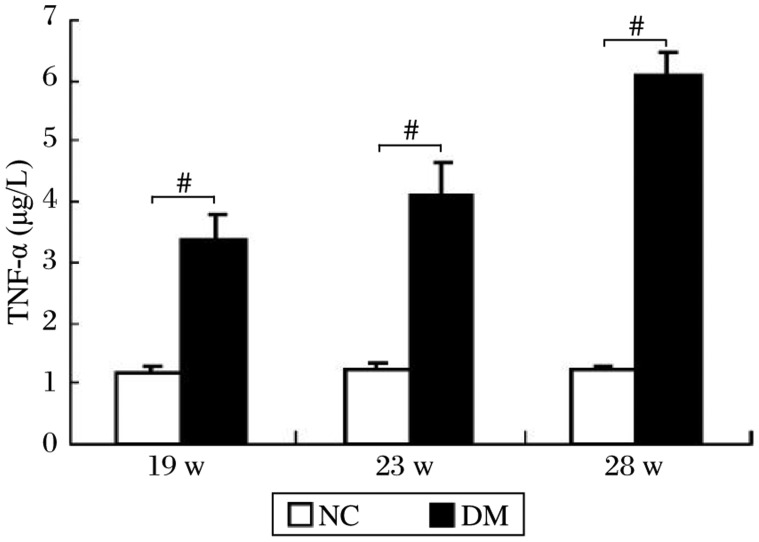
Circulating TNF-α concentration at different time points of the experiment. Data are presented as mean±SE of the mean. ^#^*P* < 0.01 compared to the normal control (NC) group.

At the 19^th^, 23^rd^ and 28^th^ week of the experiment, circulating TNF-α concentration in rats with type 2 diabetes was significantly higher than that of normal rats and showed an increasing tendency (***Fig. 6***). Significant correlation (*r* = 0.871, *P* < 0.01) was found between circulating TNF-α levels and fgl2 expression in the kidneys of diabetic rats.

## DISCUSSION

Fgl2 is a member of the fibrinogen-related protein superfamily of proteins, which has been shown to play a pivotal role in microthrombosis[9],[15]. In the present study, it was demonstrated for the first time, to our knowledge, that fgl2 as a source of procoagulant activity was abundant in the glomerular and tubulointerstitial endothelial cells of rats with type 2 diabetes and became upregulated with the deterioration of type 2 diabetes mellitus. It was identified in our findings that an association existed between the expression of fgl2 and microthrombosis in the glomerular and tubulointerstitial capillaries. Microthrombosis due to fgl2 contributed to some extent to renal impairment of rats with type 2 diabetes. Moreover, proinflammatory cytokines, such as TNF-α, could promote the expression of fgl2 in renal capillaries of rats with type 2 diabetes.

As known, diabetes is a prothrombotic state. Elevated levels of procoagulant factors increase risks for arterial thrombi in diabetic patients[16],[17]. In a morbid state, fgl2 is abundantly expressed in the microcirculation, and its expression is promoted by viral proteins and proinflammatory cytokines[18],[19]. In rats with type 2 diabetes, fgl2 was found to be highly expressed in renal capillaries, which led to renal microthrombosis and the expression was associated with TNF-α.

Emerging evidence supports the idea that inflammation is involved in the pathogenesis of diabetes[20],[21]. Adipocytes can excrete proinflammatory cytokines (IL-6 and TNF-α), which exert adverse biological effects on diabetic patients[22]. In diabetic animals, TNF-α level was elevated and contributed to insulin resistance[23],[24], whereas limited data is available on the effects of TNF-α on diabetic blood coagulation. Levy named the path of fgl2-promoted blood clotting as “immunity blood coagulation”[8], which means that fgl2 is expressed in microvascular endothelial cells, macrophages and other immunocytes and the expression is regulated by proinflammatory cytokines. A range of cytokines including IFN-γ, IL-2, and IL-12 as stimuli can induce the expression of fgl2[13],[18],[25]. Preliminary data indicated that fgl2 transcription in endothelial cells occurred in response to TNF-α[26]. In experimental cytokine-induced fetal loss or viral hepatitis, TNF-α was demonstrated to promote fgl2 expression in endothelial cells of trophoblasts and decidua or in hepatic endothelial cells[12],[25]. It indicated that TNF-α was able to induce fgl2 expression in graft vascular endothelial cells, which enhanced the activation of human prothrombin and contributed greatly to acute vascular xenograft rejection[6]. In our study, levels of TNF-α in rats with type 2 diabetes were significantly elevated and were closely related to fgl2 expression. Based on the previous studies and our results, we concluded that TNF-α promoted fgl2 expression in glomerular and tubulointerstitial endothelial cells of rats with type 2 diabetes.

Fibrin deposition in microvessels leading to hyaline microthrombosis in situ was demonstrated due to fgl2 expression[6],[10],[27]. Activated fgl2 initiates microthrombosis by directly cleaving prothrombin to thrombin, which does not act in the same way as the classic extrinsic and intrinsic coagulants do[8],[10]. Our results showed that fgl2 and consequent hyaline microthrombosis were observed at renal microvessels in diabetic rats, which suggests that fgl2 may play a role in renal microangiopathy of rats with type 2 diabetes.

The possible pathogenetic mechanisms involved in diabetic nephropathy are still inconclusive. Chronic hyperglycemia is the main cause of diabetic nephropathy. Multiple theories have been proposed to explain the adverse effect of hyperglycemia, including an increased flux through the polyol pathway, excessive formation of advanced glycation end-products, oxidative stress, and activation of the protein kinase C pathway[28]–[31]. In addition to metabolic factors, high intraglomerular pressure and high ultrafiltration cause a heavy burden on the filtration network and aggravate diabetic glomerular injuries. In general, the metabolic and hemodynamic stress can trigger thickening of the GBM and expansion of the mesangium due to accumulation of extracellular matrix production, which is fundamental in the morphological abnormalities of diabetic nephropathy[4],[32] and was also observed in rats with type 2 diabetes. Nevertheless, the mechanism by which diabetes induces glomerular hypertension is still being defined[32],[33]. In this study, glomerular microthrombi as a novel trigger might precipitate glomerular hypertension and glomerulopathy. The direct effect of sporadic microthrombosis in glomerular capillaries was microvascular obstruction and pressure alteration. The obstruction due to microthrombi located away from the afferent arteriole increased blood flow resistance and resulted in local glomerular hypertension, thereby causing glomerular capillary expansion. Additionally, the capillaries following obstructive microthrombi became collapsed, thereby resulting in ischemia. In addition, tubulointerstitial microthrombosis contributed to renal ischemia.

In summary, this study reports the novel finding that fgl2 can act as an activator of renal microthrombi contributing to glomerular hypertension and renal ischemia, which can trigger histologic alterations in experimental type 2 diabetic nephropathy. Our study provides novel insights into the pathophysiological mechanism of diabetic nephropathy. Further studies are warranted to elucidate whether specifically inhibiting fgl2 activation or downregulating fgl2 expression could delay the progression of diabetic nephropathy.
